# **Simple Synthesis and Biological Evaluation of Some Benzimidazoles Using Sodium Hexafluroaluminate, Na**_**3**_ **AlF**_**6**_ **, as an Efficient Catalyst**

**Published:** 2014

**Authors:** Akbar Mobinikhaledi, Ahmad Hamta, Mehdi Kalhor, Mehdi Shariatzadeh

**Affiliations:** a *Department of Chemistry, Faculty of Science, Arak University, Arak 38156-8-8349, Iran.*; b *Department of Biology, Faculty of Science, Arak University, Arak 38156-8-8349, Iran.*; c *Department of Chemistry, Faculty of Science, Payam Noor University.*; d *Department of Biochemistry, Shiraz University, Shiraz, Iran.*

**Keywords:** Aromatic aldehydes, Bbenzimidaazole, Catalyst, *o-*Phenylenediamine

## Abstract

Considerable attention has been focused on the synthesis of benzimidazoles due to having a broad spectrum of biological activities such as anti-parasitic, fungicidal, anti-thelemintic and anti-inflammatory activities. As a part of our research work in this area, a series of benzimidasole derivatives (3a-n) were synthesized in good to high yields by reaction of *o*-phenylenediamine and different aromatic aldehydes in the presence of sodium hexafluroaluminate, Na_3_AlF_6_, as an efficient catalyst at 50 ^◦^C. This environmentally benign and practical method offers several advantages, such as high yields, use of available catalyst, mild reaction conditions and easy workup. The antibacterial activity of these benzimidasoles was also evaluated using Staphylococcus aureus (mm) and Escherichia coli (mm) bacterial strain. All synthesized materials were characterized using IR and NMR spectroscopy as well as microanalyses data.

## Introduction

Benzimidazole and imidazole derivatives are very useful bioactive intermediates for the preparation of pharmalogical and biological active molecules (1-6). This ring system is present in numerous antiparasitic, fungicidal, antithelemintic and anti-inflammatory agents (4-7). There are two general methods for the synthesis of 2-substituted benzimidazoles (8-19). The most general method is a condensation reaction of *o*-phenylenediamine with carboxylic acids or their derivatives such as nitriles, imidates or ortho esters (10), under strongly acid conditions or in combination with very high temperatures and/or microwave irradiations. The other one includes an oxidative cylocondensation reaction of Schiff bases in turns by condensation of *o*-phenylenediamine with aldehyde (6, 8, 9). A variety of oxidants and catalysts have been used for preparation of benzimidazoles. Although these methods worked nicely in many cases, however, some of these suffer from one or more limitations such as low yields, use of volatile or toxic organic solvents, requirement of excess amounts of catalysts or reagents, special apparatus and harsh reaction conditions. Consequently, development of convenient, high yield and environmentally benign procedure for synthesis of benzimidazoles is still a challenging research.

Sodium hexafluroaluminate (Na_3_AlF_6_), known as cryolite, is a non-toxic and commercially available compound that can be used as a catalyst in the laboratory without special precautions.

Due to the important biological activity of benzimidazoles and in line with our research works in synthesis of this ring system (20-24) we wish to report a simple procedure for preparation of 2-arylbenzimidazoles through a condensation reaction of *o*-phenylenediamine and aromatic aldehydes in the presence of Na_3_AlF_6_ as a catalyst. We also report the antibacterial activity of these compounds.

## Experimental

Melting points were determined on an electrothermal digital melting point apparatus and are uncorrected. The IR spectra were recorded on Unicom Galaxy series FT-IR 5000 spectrometer. NMR spectra were recorded on a Bruker Avance (300 MHz) spectrometer. Chemical shifts (ppm) were referenced to the internal standards tetramethylsilane (TMS). Microanalyses were performed by the Elemental Analyzer (Elemental, Vario EL III). The Microanalyses results were agreed favorably with the calculated values. Reactions were monitored by thin layer chromatography using silica gel F_254_ aluminum sheets**.** Almost all synthesized compounds are known and identified using IR and NMR spectroscopy and also by comparison with their authentic samples.

*General procedure for preparation of 2-arylbenzimidazoles* *(3a-n)*

To a solution of *o*-phenylenediamine (1 mmol) and corresponding aromatic aldehyde (1 mmol) in ethanol (20-25 mL) was added Na_3_AlF_6_ (2 mol%). The reaction mixture was stirred at 50 C for desired time. The progress of reaction was monitored by TLC. After completion of reaction for appropriate time (Table 3), water (3-40 mL) was added to give the crystals, which then filtered and washed with cold water and air dried. 

*Antibacterial study*

We used the agar disk diffusion test or Kirby-Bauer disk-diffusion method. The microbial strains are identified strains and were obtained from the Pasteur Institute of Iran. The bacterial strains studied are Staphylococcus aureus (RTCC, 1885), and Escherichia Coli (ATCC, 35922). Each chemically synthesized material (5 mg) was dissolved in 250 µL of DMSO (20 µg/µL) and 100 µL of the solution of the test compounds was introduced onto the disks (0.7 cm diameter). The disks were then placed on top of the medium previously inoculated with bacteria. 100 µL of solvent (DMSO) was added to another disk and implanted as a negative control on each plate along with the standard drugs. The plates were incubated overnight at 37 °C. The inhibition zones were measured and compared with the standard drugs. For the zone size interpretations were used recommendations of the National Committee of Clinical Laboratory Standards (NCCLs). The results are given in Table 4. The inhibition zone numbers are the average of three times independent experiments.

*2-Phenyl-1H-benzimidazole (3a)*

IR (KBr): ν= 3348 (NH), 3047 (CH_aromatic_), 1462 (C=C) cm^-1^. ^1^HNMR (300 MHz, DMSO): δ ppm = 7.20-7.65 (m 7H, CH_aromatic_), 8.16-8.20 (m, 2H, CH_aromatic_), 12.93 (bs, 1H, NH). Anal. Calcd. For C_13_H_10_N_2_: C, 80.39; H, 5.19; N, 14.42%. Found: C, 80.58; H, 5.40; N, 14.51%.

*2-(4-Methylphenyl)-1H-benzimidazole (3b)*

IR (KBr): ν = 3323 (NH), 3059 (CH_aromatic_) 1448 (C=C), 1622 (C=N) cm^-1^. ^1^HNMR (300 MHz, DMSO): δ (ppm) = 2.38 (s, 3H, CH_3_), 7.17-8.08 (m, 8H, CH_aromatic_), 12.83 (bs, 1H, NH). Anal. Calcd. For C_14_H_12_N_2_: C, 80.74; H, 5.81; N, 13.45%. Found: C, 80.71; H, 6.02; N, 13.39%.

*2-(2-Nitrophenyl)-1H-benzimidazole (3c)*

IR (KBr): = 3364 (NH), 3024 (CH aromatic), 1446 (C=C), 1518 (N=O) cm^-1^. ^1^H NMR (300 MHz, acetone-d_6_): δ (ppm) = 7.25-8.05 (m, 8H, aromatic), 13.06 (bs, 1H, NH). Anal cald for C_13_H_9_N_3_O_2_: C, 65.27; H, 3.79; N, 17.56%. Found: C, 65.01; H, 3.96; N, 17.74%.

*2-(3-Nitrophenyl)-1H-benzimidazole (3d)*

IR (KBr): = 3358 (NH), 3086 (CH aromatic), 1431 (C=C), 1516 (N=O) cm^-1^. ^1^H NMR (300 MHz, acetone-d_6_): δ (ppm) = 7.28-9.05 (m, 8H, aromatic), 12.21 (bs, 1H, NH). Anal cald for C_13_H_9_N_3_O_2_: C, 65.27; H, 3.79; N, 17.56%. Found: C, 65.51; H, 3.59; N, 17.63%.

*2-(4-Nitrophenylhenyl)-1H-benzimidazole (3e)*

IR (KBr): ν = 3367 (NH), 3061 (CH_aromatic_) 1516 (C=C), 1597 (C=N) cm^-1^. ^1^HNMR (300 MHz, DMSO): δ ppm = 7.254-7.75 (m, 4H, CH_aromatic_), 8.39 (bs, 4H, CH_aromatic_), 13.30 (bs, ^1^H, NH). Anal. Calcd. For C_13_H_9_N_3_O_2_: C, 65.27; H, 3.79; N, 17.56%. Found: C, 65.39; H, 3.58; N, 17.47%.

*2-(4-Bromophenyl)-1H-benzimidazole (3g* **)**

IR (KBr): ν = 3350 (NH), 3051 (CH_aromatic_) 1429 (C=C) cm^-1^. ^1^HNMR (300 MHz, DMSO): δ 7.20-8.12 (m, 8H, CH_aromatic_), 13.0 (bs, 1H, NH). Anal. Calcd. For C_13_H_9_BrN_2_: C, 57.17; H, 3.32; N, 10.26%. Found: C, 57.23; H, 3.52; N, 10.34%. 

*2-(2-Chlorophenyl)-1H-benzimidazole (3h)*

 IR (KBr): ν = 3377 (NH), 3061 (CH_aromatic_) 1440 (C=C), 1599 (C=N) cm^-1^. ^1^HNMR (300 MHz, DMSO): δ ppm = 7.21 (q, J= 2.6 Hz, 2H, CH_aromatic_), 7.501-7.54 (m, 3H, CH_aromatic_), 7.66 (q, J= 3.8 2H, Hz, CH_aromatic_) 7.90 (t, ^1^H, J= 9.4 Hz, CH_aromatic_), 12.73 (bs, 1H, NH). Anal. Calcd. For C_13_H_9_N_2_Cl: C, 68.28; H, 3.97; N, 12.25%. Found: C, 68.40; H, 4.05; N, 12.38%.

*2-(3-Chlorophenyl)-1H-benzimidazole (3i)*

IR (KBr): = 3354 (NH), 3045 (CH aromatic), 1442 (C=C), 744 (C-Cl). ^1^HNMR (300 MHz, acetone-d_6_): δ ppm = 7.24-8.27 (m, 8H, aromatic), 12.00 (bs, ^1^H, NH). Anal cald for C1_3_H_9_N_2_Cl: C, 68.28; H, 3.97; N, 12.25%. Found: C, 68.07; H, 4.10; N, 12.03%.

*2-(4-Chlorophenyl)-1H-benzimidazole (3j)*

IR (KBr): = 3342 (NH), 3055 (CH aromatic), 1448 (C=C), 746 (C-Cl) cm^-1^.^ 1^HNMR (300 MHz, acetone-d_6_): δ ppm = 7.18-8.21 (m, 8H, aromatic), 13.00 (bs, ^1^H, NH). Anal cald for C_13_H_9_N_2_Cl: C, 68.28; H, 3.97; N, 12.25%. Found: C, 68.03; H, 4.21; N, 12.41%.

*2-(2-Hydroxy-5-bromophenyl)-1H-benzimidazole (3k)*

IR (KBr): ν = 3364 (NH), 3065 (CH_aromatic_) 1436 (C=C) cm^-1^. ^1^HNMR (300 MHz, acetone-d_6_): δ ppm = 5.31 (s, 1H, NH), 6.75-7.54 (m, 7H, aromatic), 10.28 (bs, ^1^H, NH). Anal cald for C_13_H_9_N_2_BrO: C, 54.00; H, 3.14; N, 9.69%. Found: C, 54.23; H, 3.36; N, 9.82%.

*2-(3-Metoxyphenyl)-1H-benzimidazole (3l)*

IR (KBr): ν = 3051 (CH_aliphatic_), 2924 (CH_aromatic_), 1602 (C=N), 1464 (C=C) cm^-1^. ^1^HNMR (300 MHz, DMSO): δ 3.07 (s, 3H, OCH_3_), 7.46-8.22 (m, 8H, CH_aromatic_) 13.30 (bs, ^1^H, NH). Anal. Calcd. For C_14_H_12_N_2_O: C, 74.98; H, 5.39; N, 12.49%. Found: C, 75.10; H, 5.31; N, 12.59%. 

*2-(4-Metoxyphenyl)-1H-benzimidazole (3m)*

IR (KBr): ν = 3481 (NH), 3132 (CH_aromatic_) 1437-1502 (C=C), 1622 (C=N) cm^-1^. ^1^HNMR (300 MHz, DMSO): δ ppm = 3.82 (s, 3H, OCH_3_), 7.11-7.17 (m, 2H, CH_aromatic_), 7.21 (q, J= 3.2 Hz, 2H, CH_aromatic_), 7.54 (t, J= 3.0 Hz, 2H, CH_aromatic_), 8.16 (t, J= 8.9 Hz, 2H, CH_aromatic_) 12.75 (bs, 1H, NH). Anal. Calcd. For C_14_H_12_N_2_O: C, 74.98; H, 5.39; N, 12.49%. Found: C, 74.79; H, 5.46; N, 12.29%.

*2-(3,5-Dimethoxyphenylhenyl)-1H-benzimidazole (3n)*

IR (KBr): ν = 2849 (CH_aliphatic_) 3055 (CH_aromatic_) 1504 (C=C), 1606 (C=N) cm^-1^. ^1^HNMR (300 MHz, DMSO): δ 3.84 (s, 3H OCH_3_), 3.894 (s, 3H OCH_3_), 7.12-7.77 (m, 7H, CH_aromatic_), 12.76 (bs, 1H, NH). Anal. Calcd. For C_15_H_14_N_2_O_2_: C, 70.85; H, 5.55; N, 11.02%. Found: C, 70.96; H, 5.41; N, 11.12%.

## Results and Discussion

**Figure 1:**
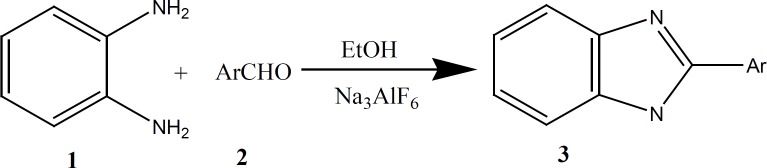
The synthetic pathway of benzimidazoles in the presence of sodium hexafluroaluminate

Reactions were carried out by taking a 1:1 mol ratio mixture of *o*-phenylenediamine with aromatic aldehydes 2 in the presence of Na_3_AlF_6_ in ethanol to give 2-arylbenzimidazoles (Figure 1). However, aliphatic aldehydes such as formaldehyde or acetaldehyde were also tested under the same conditions, but the corresponding products were isolated in trace amounts. 

For optimization of the amount of catalyst required for this reaction,* p*-*nitrobenzaldehyde* was used as a model compound and different amounts of catalyst were tested under the same conditions. It was found that 2 mol% of catalyst was enough for a fairly high yield (Table 1). On the other hand, an amount of catalyst over than 2 mol% did not improve the yield of product.

**Table 1:** Reaction of* o-*phenylenediamine with *p*-*nitrobenzaldehyd* in ethanol using different amounts of catalyst at 50  C.

**Entry**	**Mol% Catalyst**	**Time (h)**	**Yield (%)**
1	2	2	80
2	4	2.1	80
3	5	2.5	80
4	7	3	80

To examine the effect of solvent for this model reaction, we have also performed the reaction in various organic solvents at room temperature with 2 mol% of Na_3_AlF_6_. As Table 2 shows, ethanol is more suitable solvent for this procedure. Consequently the reaction was carried out in ethanol with 2 mol% of Na_3_AlF_6 _for the preparation of benzimidazoles (3a-n). The results are summarized in Table 3.

**Table2:** Reaction of* o-phenylenediamine* with *p-nitrobenzaldehyd* using using different solvents, prompted by 2 mol%Na_3_AlF_6_ at 50  C

**Entry**	**Solvent**	**Time (h)**	**Yield (%)**
1	Ethanol	2	80
2	Methanol	2.3	60
3	DMF	3	60
4	DMSO	3.5	64
5	Acetonitrile	3.5	44

**Table 3:** Reaction of *o*-*phenylenediamine* with aromatic aldehydes, prompted by 2 mol% Na_3_AlF_6_ in C_2_H_5_OH at 50  C

**Product (3)**	**Ar**	**Time (h)**	**Yield (%)**	**M.P. (C)**
**a**	C_6_H_5_	11	80	282-284 (290-292)^a^
**b**	4-CH_3_C_6_H_4_	9	81	264-266 (268-270)^ a^
**c**	2-NO_2_C_6_H_4_	17	68	269-271 (264-266)^b^
**d**	3-NO_2_C_6_H_4_	13	72	205-207 (203-204)^c^
**e**	4-NO_2_C_6_H_4_	2	80	310-312 (312-314)^ a^
**f**	3-BrC_6_H_4_	7.5	75	248-250
**g**	4-BrC_6_H_4_	4	92	284-296 (283-284)^b^
**h**	2-ClC_6_H_4_	15	60	231-232 (232-234)^ a^
**i**	3-ClC_6_H_4_	8	68	232-234 (234-236)^b^
**j**	4-ClC_6_H_4_	16	83	293-294 (291-293)^ a^
**k**	2-HO,5-BrC_6_H_3_	1	96	207-208 (256-257)^b^
**l**	3-OCH_3_C_6_H_4_	13	95	204-206
**m**	4-OCH_3_C_6_H_4_	14.5	80	222-224 (225-226)^ a^
**n**	3,5-(OCH_3_)_2_ C_6_H_3_	16	62	232-233

a= [ref 19], b= [ref 21], c= [ref 11]

**Scheme 1:**
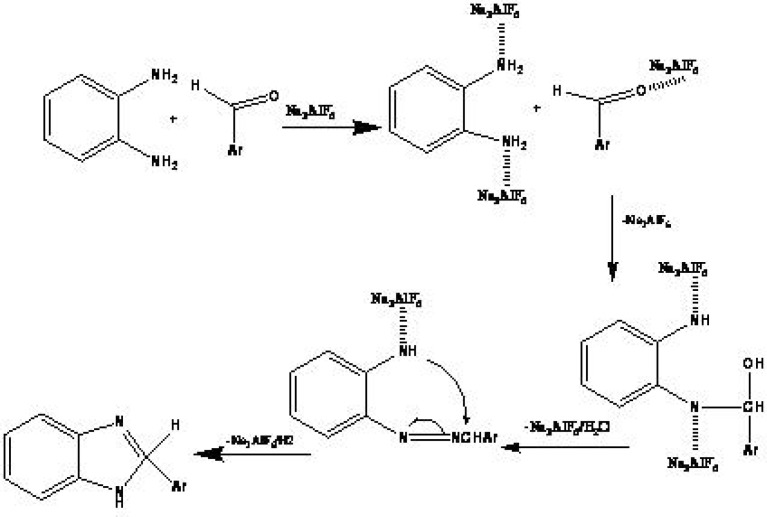
The proposed mechanism for synthesis of benzimidazoles in the presence of sodium hexafluroaluminate as a catalyst

A possible mechanism, supported by literatures (25), has proposed for this reaction. The reaction may prompt by an interaction between the carbonyl group and Na_3_AlF_6_ as shown in Scheme 1. The ^1^H and ^13^C NMR spectra as well as the elemental analyses data of all synthesized compounds are consistent with the expected structures. The ^1^H NMR spectra of benzimidazoles (3a-n) consists of a multiplet and a broad singlet at downfield shift resulting from the aromatic protons and the NH group, respectively. 

The investigation of antibacterial screening data revealed that the compounds 3e, 3j, 3k and 3n show antibacterial activities against *E. coli*, as gram negative bacteria (Table 4). Also compounds 3a, 3b, 3e, 3i, 3j, 3k, 3l and 3n, showed good inhibition against *S. aureus* as compared to penicillin zone of inhibition. The anti-*S. aureus* activity of compounds 3j, 3k and 3n with inhibition zone of 36, 43 and 32 mm, respectively, are better than that of other compounds.

**Table 4:** 1 Zone inhibition of benzimidazoles (3a-n).

**Compound (3)**	***Staphylococcus aureus*** ** (mm)**	***Escherichia coli*** ** (mm)**
**a**	21	–
**b**	23	–
**c**	–	–
**d**	–	–
**e**	18	13
**f**	-	–
**g**	-	–
**h**	–	–
**i**	20	–
**j**	36	14
**k**	43	27
**l**	16	–
**m**	–	–
**n**	32	10
**DMSO**	–	–
**Standard drugs**	Penicillin 33 mm	Gentamicin 18 mm

–: indicates bacteria are resistant to the compounds.

Zone of inhibition are reported in mm of diameter.

Discs were inoculated with 5 mg of the compounds dissolved in DMSO.

In conclusion, we have developed a simple and high efficient procedure for the synthesis of 2-arylbenzimidazoles with advantages of operational simplicity, good to high yields and use of non-toxic and commercial available catalyst.
